# Multiplexed strategies toward clinical translation of extracellular vesicles

**DOI:** 10.7150/thno.75899

**Published:** 2022-09-21

**Authors:** Junying Song, Baoqiang Song, Lijun Yuan, Guodong Yang

**Affiliations:** 1 State Key Laboratory of Cancer Biology, Department of Biochemistry and Molecular Biology, Fourth Military Medical University, Xi'an, 710032, People's Republic of China.; 2 Department of Plastic and Reconstructive Surgery, Xijing Hospital, Fourth Military Medical University, Xi'an, 710032, People's Republic of China.; 3 Cadet Team 6 of School of Basic Medicine, Fourth Military Medical University, Xi'an, People's Republic of China.; 4 Department of Ultrasound Diagnostics, Tangdu Hospital, Fourth Military Medical University, Xi'an, 710038, People's Republic of China.

**Keywords:** extracellular vesicles, clinical translation, yield, isolation, cargo loading, delivery, manufacturing

## Abstract

Extracellular vesicles (EVs), of which exosomes are a representative subgroup, are naturally secreted nanoparticles with a variety of payloads. With the intrinsic merits of stability, biocompatibility, low immunogenicity, and large capacity, EVs are widely regarded as effective carriers of drug delivery. However, disadvantages, such as low yield, complicated isolation procedures, and low loading efficiency, hinder its clinical translation. In this review, we systematically summarize the advances in EV (especially exosomes) engineering for clinical application, focusing on strategies toward high yield, facile isolation, efficient cargo loading, improved delivery, and optimized manufacturing, which might unleash the infinite power of EVs in clinical translation.

## Introduction

Nanomedicine-based theranostics has significantly impacted conventional therapeutic modalities. Rapid developments in nanotechnology have allowed us to synthesize nanomaterials with designed properties, such as electromagnetism, targeting ability, and thermal responsiveness, enabling clinicians to treat diseases precisely 1. Drug delivery is at the core of clinical therapeutics. Due to the large ratio of surface area to volume, excellent biocompatibility, and prolonged circulation time, artificially assembled nanoparticles are considered a fascinating carrier, bringing about revolutionary changes in treatment. Nevertheless, the blockade effect from physiological barriers and unwanted uptake by the reticuloendothelial system impede the application of exogenous nano-systems 2, 3. Conventional viral vectors used in gene therapy also have limited application due to oncogenicity, immunogenicity, and intrinsic pathogenicity 4. Scientists are, therefore, actively seeking a promising substitute with superior performance.

Extracellular vesicles (EVs), including microvesicles (MVs) and exosomes or exosome-like vesicles (ELVs), have been used as effective carriers in drug delivery with remarkable capability to extravasate through vessel fenestrations and move through extracellular matrix 5. The focus of this review is on exosomes, a major subgroup of EVs. As organelles of ~30 to ~200 nm in diameter and 1.08 to 1.22 g/mL in density 6, 7, exosomes are budded from the endosome membrane and endowed with a variety of distinctive features, playing a significant role in multiple biological processes 8, 9. Previous studies have unraveled that exosomes can cross the blood-brain barrier 10 and maternal-placental barrier 11. The phospholipid bilayer provides excellent membrane permeability and protects the encapsulated agents from degradation 12. Modes of administration for EVs range from nasal and oral administration to stereotactic and intravenous injection, rendering options for various diseases 13. EVs have shown unprecedented potential in diagnostics and therapeutics 14-16.

Clinical translation of EVs for drug delivery appears to be within reach. Preclinical data published by Codiak BioSciences Inc. demonstrated the potent anti-neoplastic activity of the candidate product exoASO-STAT6 17. Also, several pharmaceutical products have already entered human trials, such as Plexaris and Cevaris of ExoPharm and CAP-2003 of Capricor Therapeutics. Currently, many companies dedicated to cell therapy are diligently developing EV products for clinical applications 16, 18. However, several bottlenecks of productivity, biopotency, and biosafety remain and must be resolved. Yuan et al. proposed the “STOP” criterion that refers to high Safety, good Targeting ability, rapid Obtainment, and high Purity 19. Unlike laboratory experiments, there is still a huge gap between inadequate yield and sustainable medical demands. Additionally, the inefficiency of cargo encapsulation has restricted the progress of EV application 20. Upgrading engineering strategies, isolation methods, and introducing well-designed modifications to EVs, will undoubtedly promote their clinical application.

**Figure 1:**
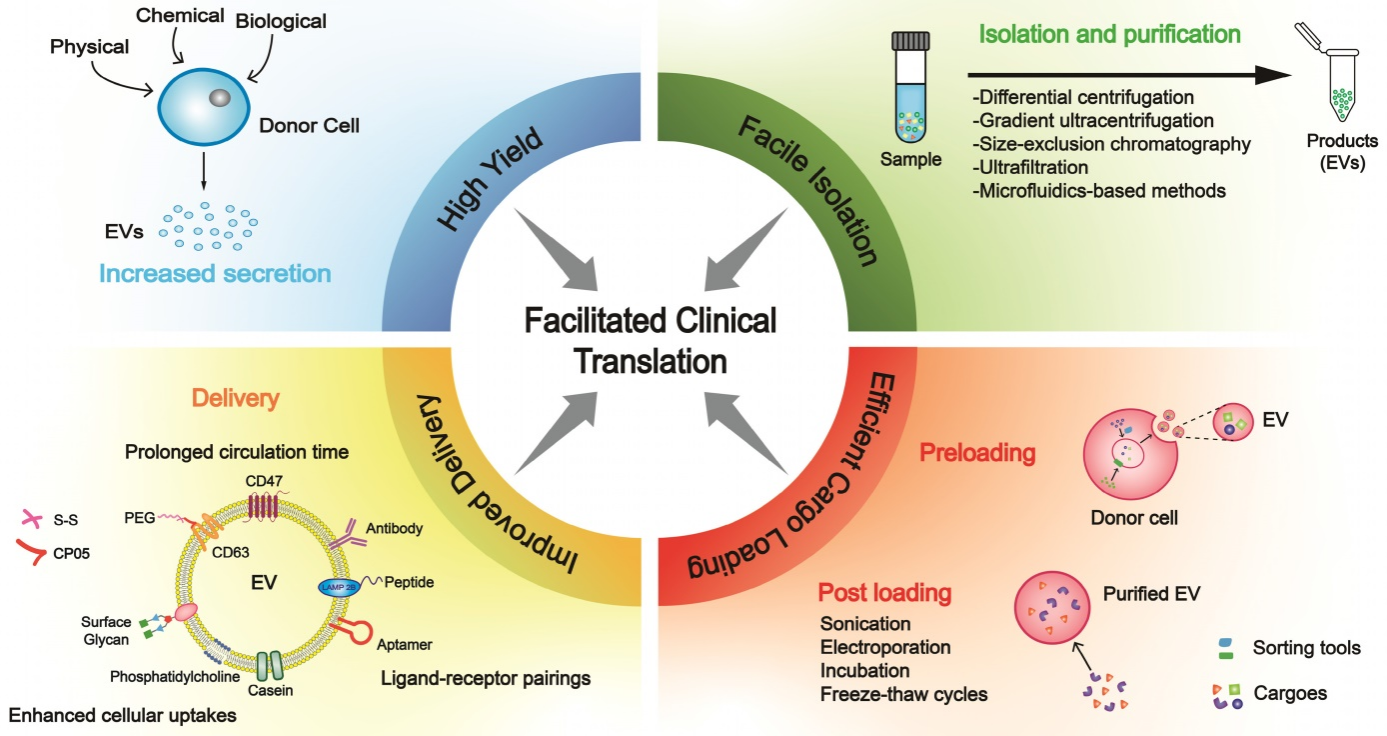
** Building blocks for clinical translation of EVs.** Successful application requires an integrated platform, incorporating methodologies for high yield, facile isolation, efficient cargo loading, and improved delivery. Based on diverse disease patterns, we could customize a concrete schedule for each patient to achieve personalized treatment. EV, extracellular vesicle; PEG, polyethylene glycol.

In this review, we focused on strategies in areas of high yield, facile isolation, efficient cargo loading, improved delivery, and optimized manufacturing (Figure 1), which are closely interlinked with clinical translation.

## Approaches to overcome low yield bottleneck

The history of exosomes dates back to 1983, when they were described to originate from sheep reticulocytes as released vesicles 21. However, the vesicles were not given the name “exosome” until 1987 by scientists from McGill University 22, depicting its structure vividly as “though tiny, perfectly formed”. Bioactive substances, such as proteins, lipids, and nucleic acids are encapsulated and subsequently delivered to recipient cells, eventually leading to phenotypic changes. As secreted vesicles, the biogenesis of exosomes is a finely-tuned process mediated by modulatory machinery. With the maturation of early to late endosomes, the endosomal membrane invaginates inward and transforms into a multivesicular body (MVB) 16, 23 with the support of the endosomal sorting complex required for transport (ESCRT) of complexes 0, I, II, and III 24. After the fusion between MVBs and the cellular membrane, intraluminal vesicles (ILVs) are successsfully released, which are widely acknowledged to be exosomes or ELVs 16. There are other ESCRT-independent pathways for the formation of exosomes, either triggered by the cone-shaped ceramide to generate a spontaneous negative curvature 25, 26, or marked and controlled by RAB31 to prompt epidermal growth factor receptor (EGFR) entry into MVBs 27. EVs are composed of diverse constituents, which are incorporated via sorting mechanisms such as AAUGC motif for miRNAs 28 and ubiquitin-like 3 (UBL3) modification for proteins 29. From the opposite perspective, clearing MVBs by fusion with lysosomes or autophagosomes is also a critical stage in their lifecycle 30.

**Figure 2:**
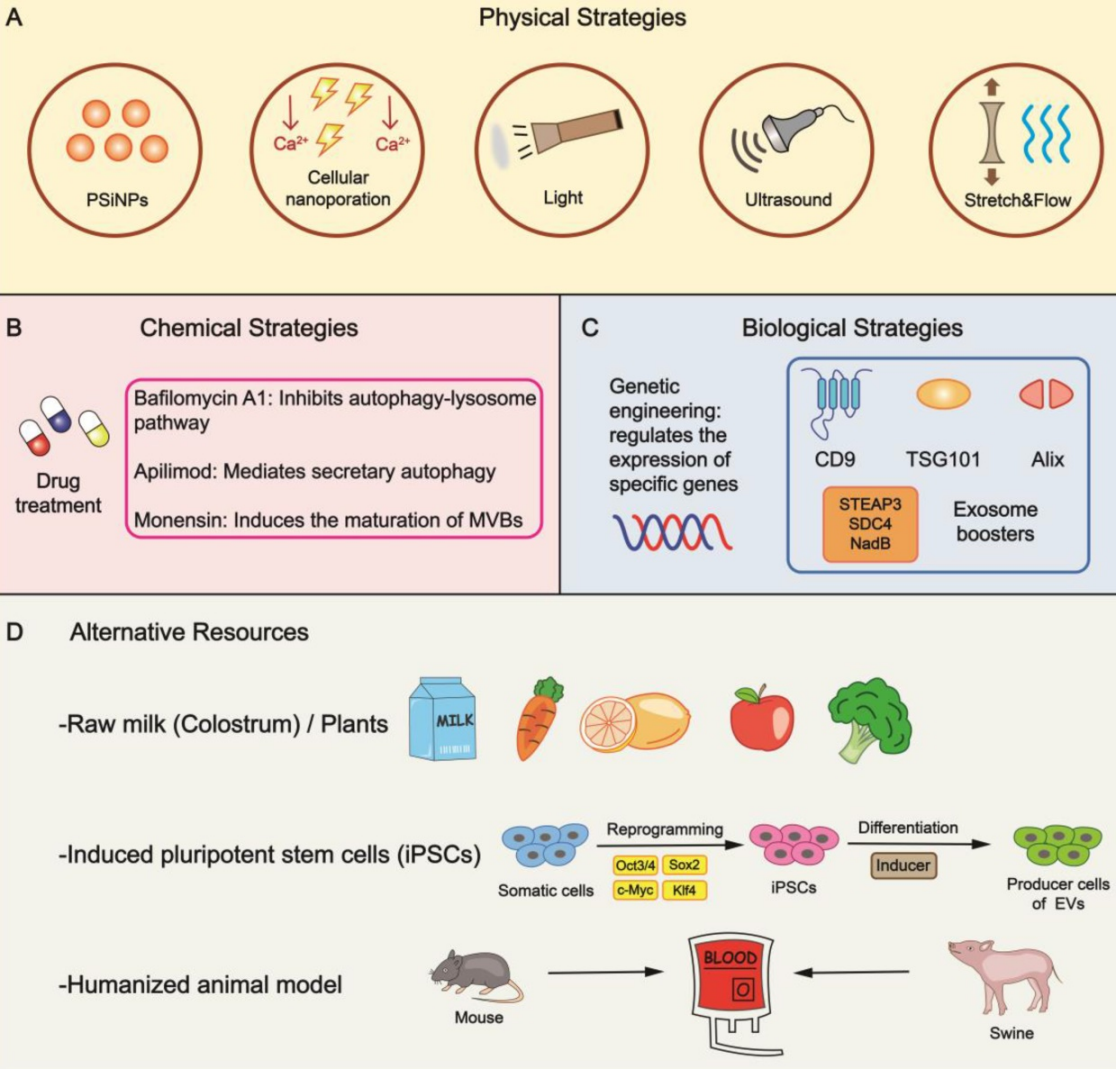
** Bioinspired strategies for increasing EV yield. (A)** Specific physical stimulations are feasible to raise the output of EVs. **(B)** The addition of drugs is a general form of chemical strategy in mass production by impacting different phases of EV biogenesis. **(C)** Altering the expression of specific genes leads to an increase in yield through genetic engineering. Exosome boosters are effective targets to be manipulated. **(D)** Several alternative means can be utilized to provide reliable resources. PSiNPs, porous silicon nanoparticles.

The quantity of naturally secreted EVs by mammalian cells is relatively low, inevitably hindering their clinical application. In this section, we will summarize promising methods to increase the biogenesis of EVs (mainly exosomes) (Figure 2).

### Physical strategies

Physical stimuli in various forms are explored to enhance EV yield. For example, tumor cells can generate a nearly 34-fold output of exosomes when incubated with porous silicon nanoparticles (PSiNPs) with a minimal effect on exosome identity 31. Mechanically, PSiNPs booster exosome biogenesis in an autophagy-dependent manner owing to their non-degradability in the lysosomal acidic microenvironment. Theoretically, the strategy should be applicable to other cells, including non-malignant cells. Yang et al. reported that by regulating the flow of calcium ions and generating electrical stimulus in a focal and transient pattern, cellular nanoporation (CNP) induced a 50-fold increase in exosome yield 32. Also, 3D scaffolds could be viewed as an innovation of traditional 2D cell cultures. Mediated by yes-associated protein (YAP) mechanosensitivity, mechanical forces remarkably fostered EV release on 3D scaffolds in the form of flow and cyclic stretch 33. Treatments such as light induction and ultrasound stimulation also led to a 13-fold and 4.14-fold increase in EV secretion respectively 34, 35. These two methods are relatively convenient to operate, but more attention should be on addressing safety and stability concerns. Physical stimuli would inevitably affect the morphology of EVs to varying degrees; therefore, ascertaining appropriate parameters (e.g., intensity, frequency, duration) is imperative.

Besides promoting biogenesis, serial extrusion is a feasible and economical approach to producing exosome-mimetics (EMs) or ELVs. Cells could be extruded by filtration with filters of specific sizes, such as titanium meshes 36, polycarbonate filters 37 and nanoporous membranes 38. The entire process averts the time-consuming course of ultracentrifugation and only requires a reasonable number of cells, avoiding time consumption and manpower for different purification procedures. This technique was upgraded by introducing iron oxide nanoparticles, and hypotonic treatment and homogenization were employed to harvest intracytoplasmic organelles as preliminary products for further processing 38. Notably, the EMs or ELVs are artificial products that share morphology and conformation with native exosomes. Also, high yield and scalability are their superior features, along with improved manufacturing technology. Wan et al. showed that components are instantaneously loaded into EMs during extrusion while certain cytosolic cargoes undergo selective sorting procedures into EVs 39. Measures to trace and identify the purity of collected EMs require further exploration as naturally secreted EVs, and endogenous components escaped from the cytosol during extrusion might be deemed as contaminants 40.

As for the clinical translation, production personnel should consider the expense and output rate balance. Proper modifications of cell culture interfaces provide a compatible environment for donor cells to grow and proliferate; hence, investments in developing advanced materials, such as a 3D production system, microporous frameworks, and multi-pronged bioreactors with a conflict-free combination of these principles might be a promising approach.

### Chemical strategies

Numerous chemically active agents have been shown to be involved in regulating the behaviors of parental cells. Hence, we can artificially change the culture conditions by applying drugs. Monensin (MON), a Na^+^/H^+^ exchanger, triggers the maturation of dilated MVBs in a calcium-dependent manner. On the negative side, dimethyl amiloride (DMA) suppresses the release of exosomes by perturbing calcium signaling 41. The autophagy-lysosome pathway (ALP) inhibitor bafilomycin A1(BafA1) induces cholesterol accumulation and the separation of MVBs from the plasma membrane 42, which can be considered as augmentation of exosomes by inhibiting autophagic degradation 43, 44. Phosphoinositide kinase PIKfyve showed a suppression effect in EV yield that could be reversed by its inhibitor apilimod 45. Chemical treatment is a simple method without complicated procedures; nevertheless, latent toxicity and appropriate dosages need to be assessed carefully.

### Biological strategies

Altering the levels of particular genes might instigate a stronger release of EVs; thus, we can control the production rate through manual interventions. Fussenegger et al. reported that specific exosome production boosters, including STEAP3, syndecan-4 (SDC4), and a fragment of L-aspartate oxidase (NadB), impact diverse stages of exosome biogenesis to raise yield 46. Upregulating CD9 promoted the secretion of exosomes, whereas Alix and TSG101 exhibited opposite effects 47. Markedly, various molecules associated with lipid metabolism play a significant role in EV secretion. Lipid transporters, such as the ATP-binding cassette (ABC) transporter A3, mediate exosome secretion via impacting lipid clustering and the biogenesis of MVB 48. Moreover, LPS/ATP stimulation resulted in increased release through inflammasome-induced cleavage of the trafficking adaptor protein RILP 28.

With advancements in transgenic technology, this method holds great promise in the clinic. Based on massive data, gene libraries, and visualization tools that can subsequently be verified under rigorous criteria, bioinformatics and multilevel omics have been extremely helpful in identifying biomarkers and molecular subtypes in various diseases, such as cancer and neurological disorders 49-51, which are applicable to the validation of targets with yield-boosting potency as well.

Accessibility is a critical factor in mass production. Studies have shown raw milk to be a scalable and cost-effective source for collecting large quantities of exosomes, while colostrum powder shows even greater potential 52. Also, we could utilize edible plants such as carrot, lemon, apple, broccoli, etc. as these plants are abundantly available sources. Derived exosome-like nanoparticles have shown significant therapeutic effects under many circumstances 53-55. A significant challenge of using these plants as donors is the underlying immunogenicity, which motivates the needs of in-depth investigations *in vivo*. Notably, the immunogenicity might be beneficial in the context of tumor vaccine and immunotherapy. In addition, oral delivery for certain diseases should be acceptable and tolerable.

Given the successful allogeneic blood transfusion within accordant blood types, human red blood cells, especially group O, seem to be ideal derivations with universal donors 56. The adequate reserves in blood banks offer a constant supply, and the absence of nuclear and mitochondrial DNA simplifies the purification 57. In some cases, we can replace autologous resources with allogeneic substitutes, which is appropriate for humanized animal models, including mice and swine, providing a prelude to scale-up production 58. Priority should be given to establishing appropriate models with stable expression of humanized genes and minimal individual differences. Remarkably, somatic cells could be reprogrammed into induced pluripotent stem cells (iPSCs), which can undergo differentiation into numerous cell types, such as mesenchymal stem cells (MSCs) and cardiac progenitor cells, providing a stable source for EVs 59-62. These approaches might be limited due to high research and development expenditure, but the cost might be acceptable if the strategy could be industrialized and commercialized. For example, once the iPSC lineages are established, it is relatively cheap to maintain the culture. The procedure has high similarity with antibody manufacturing, and investigators can also learn from the experience of the antibody industry and commercialization.

Cell derivations significantly determine the properties of EVs (Table 1). Although the immunogenic nature of EVs and biological/chemical impurities are vital, accumulating studies reveal that EVs from cell lines or other species should be tolerable. For personalized medicine, the patient-specific cell origin is preferred, whereas scalable cell lines are more appropriate for commercialization.

Physical/chemical/biological strategies have all contributed to increasing EV production (Figure 2). Rapid vesiculation enables us to deal with variable clinical environments and sustain long-term treatments, independent of just-in-time or off-the-shelf product modalities. However, from a negative perspective, it might impact the components of secreted EVs and lead to the decrease of active ingredients, either import of exogenous drugs or evasion of endogenous substances; hence, heterogeneity ought to be controlled accurately. The strategy of mass production in combination with excessive loading could ensure content level. Besides optimizing these methods, such as setting appropriate parameters and utilizing eliminable chemical reagents, screening programs are also beneficial for acquiring uniform products and reducing batch-to-batch variations. In the future, biomimetic methodologies could be employed for this purpose.

## Refinement of isolation methods

The isolation of EVs has always been a topic of interest in academic and industrial communities. Cellular components with similar size and density properties as EVs are present in culture media and biological fluids. Therefore, there is always a risk of contamination from cell debris, proteins, and apoptotic bodies 100. Moreover, different protocols of EV isolation are used by different investigators, impeding the development of a standard protocol. Facile isolation of EVs with high purity and cost efficiency is a prerequisite for clinical translation, encouraging us to design innovative procedures.

### Current methods

Recently, several methods ranging from conventional to newly-developed techniques have emerged that are summarized in Table 2.

Differential centrifugation (DC) is acknowledged as one of the most popular methods in the realm of isolation procedures. By adjusting the RPM, ingredients with different densities are either separated or discarded, while EVs are harvested in the form of pellets. It is important to establish whether EVs are in the precipitate or supernatant. Density gradient ultracentrifugation (DG UC) is employed as a form of DC using a gradient medium of sucrose or iodixanol to improve the purity of EVs. Nonetheless, the phenomena of aggregation, and contamination including high-density lipoprotein (HDL), low-density lipoprotein (LDL), or other elements will affect the purity of isolated EVs 101, 102. These two methods are commonly implemented for research purpose and account for a significant proportion (81%) of all applications for their relatively stable yield and standardized protocols 103. However, the pharmaceutical production of EVs requires high-expense investment for instruments and manpower.

The methods of size-exclusion chromatography (SEC) and ultrafiltration are based on molecular size. The stationary phase of SEC uses cross-linked porous polymers with a bead-like structure, while ultrafiltration capitalizes on membranes with various apertures. Lobb et al. suggested that the efficiency of SEC is approximately equivalent to DG 104. Notably, SEC requires extra procedures to harvest clinical-grade EVs, increasing the complexity; therefore, designing an automatic workflow may be desirable. Other methods based on precipitation and affinity appear to be promising. For example, Wang et al. capitalized on specific peptides (CLIKKPF) to capture exosomes attributed to its high affinity for phosphatidylserine on exosomal surfaces, and silica (SiO_2_) microspheres are designated sites to immobilize these peptides 105.

The development of the microfluidic-based method is a milestone in EV purification. As an interdisciplinary technique across nanotechnology and bioengineering, it focuses on constructing a microchannel system with specificity and sensitivity by utilizing external actuators and has already been applied in extracting EVs from urine, whole blood, plasma and other samples 7, 106-109. Tangential flow filtration (TFF) is an improved version of the microfluidic-based method, utilizing ultrafiltration membrane filters to collect exosomes from pre-treated supernatants after incubation and centrifugation 95. Likewise, samples can be purified through the 'dead-end' membranes along with perpendicular flow 7. An integrated system based on microfluidics has been enriched to a great extent, for which examples include acoustics and microfluidics 110, and immunomagnetic beads and microfluidics 111. Significantly, this technique can collect freshly released EVs immediately to avoid endocytosis by parental cells. Currently, portable commercial kits at a reduced cost and avoiding large stationary instruments are being invented extensively. Typical examples include miRCURY Exosome Plasma/Serum Kit, Saliva Exosome Purification Kit, and ExoQuick-Ultra 16. These products provide operators with preferable selections based on diverse working principles.

Low recovery rate and contamination induced by various methods adding to the impurity profile are major issues in EV isolation. Incorporating an interception unit into a microfluidic-based system might be beneficial in reducing the impurity of EV samples. Multifunctional instruments conducting isolation and detection synchronously are also a viable solution, enabling the EV recovery analysis in real-time. Also, purification based on yield boosting would significantly improve the proportion of purified EVs compared to contaminants.

### Assisted separation

Introducing specific devices might be a viable solution. For instance, researchers anchored superparamagnetic nanoparticles (SPMNs) onto exosomes via Tf-TfR (transferrin and transferrin receptor) interaction, labeled as SMNC-EXO. Under the impact of adjustable magnetic fields (MFs), exosomes could be effectively separated from the solution, as has been shown with reticulocyte-derived exosomes 112. This method has been optimized by attaching polyethylene glycol (PEG) spacers, shortening the time interval from extraction to acquisition to 3.5 h 19. Since the binding and disaggregation between Tf and TfR are pH-responsive, adjusting relevant conditions increased the precision of isolation and better maintained the original shape of exosomes. Other factors, such as temperature and ion strength, should also be considered 78.

In another study, a stimuli-mediated platform was exploited for EV isolation from plasma based on a synthetic copolymer (poly (N-isopropylacrylamide-co-N-acryloxysuccinimide), PNN). Thermal-mediated hydrophilic-to-hydrophobic phase transition induced EV accumulation, while the painting and removal of PNN on EVs could be manipulated 113. Recently, the chimeric nanocomposites of lactoferrin (LF)-conjugated 2,2-bis(methylol)propionic acid (MPA) dendrimer-coated magnetic nanoparticles (MNPs) (LF-bis-MPA-MNPs) achieved high purity and recovery rate of EVs. The nanocomposites utilized various interactions, including electrostatic attraction between electropositive LF and electronegative surface of EVs, physical adsorption, and biorecognition between LF and LF receptors 114. Technically, Chen et al. established the ultrafast-isolation system (EXODUS) to purify exosomes automatically by undergoing negative pressure oscillation and membrane vibration driven by double coupled harmonic oscillator 115.

Notably, for EV-based diagnostics, which starts from tiny amounts of samples, specific lab-based isolation methods such as microfluidic-based techniques appear to be practical. In contrast, methods such as DC and DG/UC, which need a large quantity of samples, might not be smart choices.

### Pipeline integrating biogenesis and isolation

Under specific circumstances, a rational and flexible combination of selected techniques is conducive to purification. For example, Watson et al. developed a strategy incorporating the bioreactor culture, TFF, and preparative-scale SEC and received desired effects, especially in lowering the contamination of ferritin 116. By integrating multiple advantages of different methods, the constructed pipeline exhibits the greatest promise to become the mainstream approach. As for each newly designed workflow, setting conflict detection programs can prompt the transition from experimental units to industrialization.

The strategies of high yield and facile isolation contribute to meeting clinical demands. It is worth investigating systematically the relative merits and compatibility in follow-up studies. Furthermore, cell culture, EV sensing, and quality control innovations would certainly be beneficial for clinical applications. The ability to automatically monitor the production, precisely booster biogenesis, and real-time isolation are urgently needed.

## Strategies to improve cargo loading

Key breakthrough in interpreting the role of EVs was made in 2007 when Valadi et al. discovered that exosomal miRNAs and mRNAs shuttled between cells 117. Since then, encapsulation of cargoes into this cell-free system has been in the spotlight. Enhancing the stability of the contents in the lumen of EVs is of prime importance, either in circulation or preservation, requiring innovations in loading methodologies. Each method has pros and cons in terms of endogenous or exogenous substances, correlating with molecular weight, functional groups, and lipophilicity. For preloading, cargoes are sorted during the biogenesis of EVs, and modulating the status of parental cells would impact the loading efficiency, while for post-loading, cargoes are directly packaged into EVs through manual interventions.

### Loading during biogenesis

**Figure 3:**
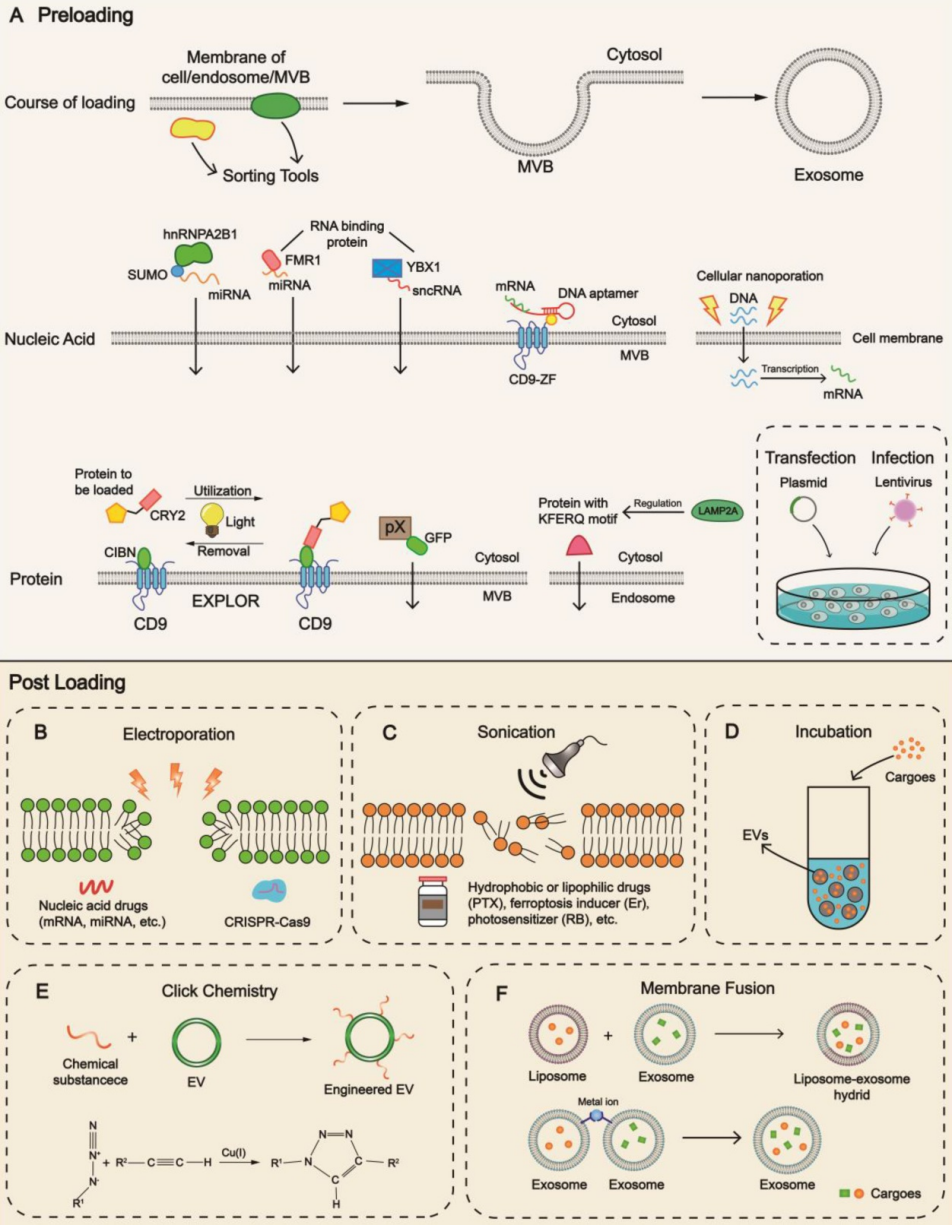
** Strategies for cargo loading into EVs.** Mechanisms and techniques for cargo loading into EVs are diverse. Preloading and post-loading are two main patterns. **(A)** Preloading is related to the sorting mechanism during biogenesis. Transfection is a frequently-used technology to increase the content level. Post-loading relies on the development of methods including **(B)** electroporation, **(C)** sonication, **(D)** incubation, **(E)** click chemistry, **(F)** membrane fusion. Different methods exhibit advantages towards different cargoes, providing cues for patient-centered clinical application. hnRNPA2B1, heterogeneous nuclear ribonucleoprotein A2B1; MVB, multivesicular body; FMR1, fragile X mental retardation 1; YBX1, Y-box protein 1; EXPLOR, exosomes for protein loading via optically reversible protein-protein interaction; CRY2, cryptochrome 2; CIBN, a truncated version of CIB1; GFP, green fluorescent protein; PTX, paclitaxel; Er, Erastin; RB, Rose Bengal; EV, extracellular vesicle.

Preloading begins before the formation of EVs with sorting mechanisms specific for different components (Figure 3A). Intracellular nucleic acids constitute a crucial branch of cargoes, and the RNA-binding protein (RBP), a constituent of the ribonucleoprotein (RNP) complex, is considered a pivotal tool to load RNA. It has been reported that fragile X mental retardation 1 (FMR1, an RBP) participated in the selective packaging of exosomal miRNA under inflammatory circumstances by recognizing specific motifs of miRNA 28. There are various reported sorting tools with nucleic acid loading ability, such as Y-box protein 1 (YBX1) 118, sumoylated heterogeneous nuclear ribonucleoprotein A2B1 (hnRNPA2B1) 119, and VAP-A along with ceramide transfer protein (CERT) 120.

Our group successfully constructed a DNA aptamer to load mRNA consisting of two segments. The single strand segment could interact with the AUG region of mRNA, suppressing mRNA translation and ribosome assembly, whereas the double strand segment could encapsulate mRNA into EVs when recognized by CD9-ZF (zinc finger) motifs 121. In another study, we fused the exosomal membrane protein CD9 with human antigen R (HuR, an RBP), facilitating miR-155 loading into exosomes specifically 122. With the emerging information on the sorting machinery of miRNA, especially the sEV-export sequences (EXOmotifs) and cellular-retention sequences (CELLmotifs) 123, it is feasible to modulate the recognition network and generate RNA-based loading by tagging them with particular motifs.

The sorting mechanism of proteins has been clarified in recent years. Choi et al. reported an excellent pattern of 'exosomes for protein loading via optically reversible protein-protein interactions' (EXPLORs) 124, allowing operators to have better control of the loading. The status of the key connector cryptochrome 2 (CRY2) is regulated via altering light conditions, changing between quiescent and photo-activated phases through phosphorylation and dephosphorylation 125. Some proteins also contain specific motifs which facilitate their loading into EVs. For example, LAMP2A mediates the sorting of proteins (e.g., hypoxia-inducible transcription factor 1, HIF1A) through the KFERQ motif pentapeptide 126. Researchers from AstraZeneca recently reported that TSPAN14, CD63, CD63TM, and CD81TM were highly efficient EV sorting proteins for loading green fluorescent protein (GFP) 127. From the engineering perspective, Kim and his colleagues constructed a truncated CD9 scaffold to load angiotensin-converting enzyme 2 (ACE2) to small extracellular vesicles (sEVs) through biogenesis, offering a binding site specific to the viral spike protein of severe acute respiratory syndrome coronavirus 2 (SARS-CoV-2) 128. It has also been reported that the structural protein pX of hepatitis A virus (HAV) facilitated eGFP (enhanced GFP) entry into MVB by interacting with the region of apoptosis-linked gene 2-interacting protein X (ALIX) 129. Future directions should concentrate on identifying more concrete domains with loading capacity.

Transfection/infection is a common technique of packaging exogenous substances into donor cells, increasing the content level and subsequently affecting the released EVs. Firefly luciferase (Fluc) and mCherry applied for EV tracking are loaded using this method 130. Technically, CNP increased the yield and encapsulated large quantities of DNA into exosomes non-genetically via creating nanochannels. Generated by electrical pulses, parental cells release plentiful exosomes with significant elevation in mRNA transcripts 32.

### Loading after biogenesis

Even after EV isolation, cargo loading is possible. In the context of loading strategies of liposomes, electroporation has been a mainstream method in encapsulating nucleic acid drugs (Figure 3B). Usman et al. confirmed that the tool for gene manipulation, clustered regularly interspaced short palindromic repeats (CRISPR)/CRISPR-associated protein 9 (Cas9) system, could be packaged into EVs through electroporation 56. This technique omits the inconvenience of designing and constructing miscellaneous vectors, inserting the cargoes into EVs via nanoscale channels directly. However, electroporation may cause the loss of endogenous components and subvert the original traits of EVs. It exhibits great potential in loading small RNAs, such as siRNA and miRNA but remains technically challenging in loading mRNA, lncRNA, or other large nucleic acid drugs. In-depth research should focus on identifying the most appropriate system and the optimal technical parameters, such as voltage and quantity of electric charge, to minimize harmful effects. Also, reducing the expense of instruments for industrial manufacturing is essential.

Sonication is another promising technique utilized in drug encapsulation of EVs by perturbing the morphology and topology of the membranes, which is better suited to hydrophobic and lipophilic cargoes (Figure 3C). Anticancer drugs such as paclitaxel (PTX), ferroptosis inducer (Erastin), and photosensitizer (Rose Bengal) can be packaged with this method 76, 131. However, without proper understanding (e.g., unfit intensity and frequency of sonic waves), an obscure alteration in physicochemical properties will result in biological dysfunction, posing a risk to safety and reproducibility.

Incubation is a spontaneous process with low efficiency 83 and is a simple method widely adopted in cargo loading of EVs, where the basic principle is passive diffusion (Figure 3D). Cargoes of interest and isolated EVs are mixed together while the duration and temperature of incubation are determined by cargo types. The membrane lipid layer of EVs accelerates the diffusion of lipophilic cargos such as fluorescent probes DiR, and DiD 130. In addition, therapeutic nucleic acids can be packaged into the liposomes to acquire lipophilicity 132. As a biosurfactant and permeabilizer, saponin can induce pore formation on the membranes by acting on cholesterol and phospholipids 133, 134. EVs incubated with saponin have been reported to exhibit a higher encapsulation percentage of hollow gold nanoparticles (HGNs) than electroporation 135.

Besides effectively producing EV mimetics, serial extrusion also has a great potential in cargo packaging, such as synthetic materials like gold nanoparticles (AuNPs) 136 and chemotherapeutic drugs like PTX 137. Extruders and filters with different diminishing pore sizes are the building blocks of this method. With repeated execution of the procedure, the dimensions of the particles can be manipulated. However, mechanical force may pose a threat to the quality and stability of EVs in subtle ways. Another technique in cargo loading of EVs is freeze-thaw cycles. For example, Haney et al. successfully loaded catalase into exosomes using this technique for treating Parkinson's disease 138. Other methods like thermal shock for HGNs, hypotonic dialysis for porphyrins, and the pH gradient method for nucleic acids have also been explored 135, 139-141.

The introduction of lipids benefits the loading of EVs. Lipids, such as cholesterol, have membrane-anchoring properties through hydrophobic interactions with the phospholipid layer. Guo et al. attached arrow-shaped RNA to EVs and altered its function by switching cholesterol positions, either in the arrowhead or arrow tail 142.

After it was first introduced by Sharpless in 2001 143, click chemistry has been broadly adopted in biomedical materials with high atom economy and was utilized for EV engineering (Figure 3E). Substrates, such as functional groups of primary metabolites, can be synthesized and conjugated through versatile crosslinkers into many products with anticipated characteristics 144. For instance, ultrasmall Prussian blue nanoparticles (uPB) for mitigating oxidative stress 145 can be decorated onto exosomes through copper-free click chemistry.

Membrane fusion is a viable strategy in fabricating engineered EVs, which could be generated between liposomes and EVs or with two individual EVs. The evolution of liposomes has exceeded half a century since Bangham first proposed it in 1965 146. Because of their distinctive conformation and optimized pharmacodynamics, liposomes have been extensively applied and commercialized in drug delivery 3. The selective packaging of cargoes into exosomes can be achieved through the fabrication of exosome-liposome hybrids (Figure 3F). For example, Li and his coworkers used biohybrids encapsulated with triptolide (TP) and miR497 for the treatment of ovarian cancer 147. Multiple methods can be implemented to construct exosome-liposome hybrids, including freeze‑thawing, co‑extruding, and incubation 40. Native exosomes may lack specific therapeutic agents or sufficient content levels. On the contrary, biohybrids (semi-artificial exosomes) can be modified with desired physicochemical features for which the technology is relatively well-established, offering better chances to carry a variety of payloads. However, before its application in clinical settings, the identity of the exosome-liposome hybrid should be established. It is important to determine whether the hybrid is more like an exosome or a liposome, whether the stability, biocompatibility, and homing property of exosomes are sacrificed during their formation, and how to achieve balanced effects with improved loading and acceptable biocompatibility/kinetics. Remarkably, the fusion between exosomes can be controlled and mediated by supramolecular complexation. Scientists assembled multiple enzymes within different engineered exosomes into a minimal electron transport chain (an unprecedented cargo type) and innovatively formed biocatalytic cascades to produce ATP with the chemical bridging method 148.

With profound technological advances, numerous methods have emerged to enhance loading capacity and the categories of cargo have been broadened. Efficient cargo loading elevates the level of agents within a single EV, lowering the amount of EVs needed, promoting therapeutic efficiency, and significantly benefiting clinical translation. In addition, processed EVs comprise a heterogenous population, and it is imperative to eliminate the unloaded fraction to raise the safety and quality level.

## Multifaceted routes towards improved delivery

Biosafety is an uncompromising benchmark in clinical practice as chemical reagents act on the human body. Furthermore, improved EV delivery should start from reducing non-specific cellular uptakes, promoting interactions with recipient cells and retention in targeted organs, and enhancing internalization efficiency (Figure 1).

### Strategies to prolong half-life period *in vivo*

Reducing the off-target cellular uptake is critical to prolonging circulation time, minimizing the needed doses, and facilitating delivery in target cells. Although the nanoscale framework prevents EVs from phagocytosis by the mononuclear phagocyte system (MPS) in the spleen and liver to a certain degree 5, they are distributed and degraded primarily in these organs 149. EVs need to be camouflaged to avoid uptake by untargeted tissues. CD47 is a well-known target in tumor immunology, which binds with the signal regulatory protein alpha (SIRPα, also known as CD172a) on macrophages, sending the signal of 'don't eat me'. CD47-SIRPα pathway is a classical immune checkpoint, permitting cancer cells to escape from immune system surveillance in the first place. This approach has been exploited to assist EVs in evading MPS by constructing corresponding plasmids to transfect the donor cells 131, 150. Moreover, knockdown of specific endocytosis-associated genes in macrophages and injection of blocking agents such as siClathrin efficiently reduced endocytosis 151. This methodology is conceptually feasible; nevertheless, its clinical prospect remains unproven. The technology of gene knockdown in humans is still challenging as the accuracy and latent mutations are difficult to control. MPS is a vital defense mechanism in pathological states, and its blockade may lead to immune response disorders. Also, Clayton et al. discovered that complement regulators CD55 and CD59 anchored on exosomal glycosylphosphatidylinositol (GPI) protected the exosomes from complement-mediated lysis, effectively extending their half-life 152. Genetic engineering would enable us to apply similar modalities to the EVs of various origins.

Surface remodeling is believed to enhance the stability of EVs. PEG is a protective hydrophilic polymer and could be decorated onto EVs through specific peptides. Its high sensitivity to reactive oxygen species (ROS) results in the breakage of thioketal bonds between the peptide and PEG, leading to the removal of the outer coating of PEG, exposing the core unit (the entity of EV). The PEG corona (PEGylation) for EVs is conducive to avoiding aggregation, opsonization, and phagocytosis 153. Exosome polymer hybrids based on photo-mediated atom transfer radical polymerization have also been proven feasible to increase blood cycling time as the grafted monomers included oligo (ethylene oxide) methacrylate (OEOMA), carboxybetaine methacrylate (CBMA) and poly(2-(methylsulfinyl) ethyl acrylate) (pMSEA) 154. Strategies to maintain biological features of intrinsic exosomal surface molecules after manufacturing would be the focus of further research. Simplifying procedures and shortening reaction time (e.g., application of catalyst) might be workable solutions.

Overall, these methods significantly improve the pharmacokinetics of EVs and enhance their retention in circulation, while pre-sealed strategy of PEGylation holds great potential along with the advances in materials science and “grafting” technologies 154. Nonetheless, its clinical prospect has yet to be determined as excessive usages of PEG will give rise to the generation of anti-PEG antibodies and even severe hypersensitivity reactions. The non-biodegradability of PEG should not be neglected as well, urgently encouraging the development of alternatives with superior biocompatibility 155-157.

### Enhanced target-specific delivery and recognition by recipient cells

Scientists have discovered and utilized a wide range of pairing mechanisms based on the interaction between ligands and receptors (listed in Table 3). Several cell types possess intrinsic specificities, such as the inflammation-tropism capacity of M2 macrophages 158 and the tumor-homing property of neutrophils 71, 145, and the derived EVs could inherit these properties. Jang et al. adopted lymphocyte function-associated antigen (LFA-1) to match with overexpressed ICAM1 (a type of cell adhesion molecule) of endothelial cells in abnormal angiogenesis 37. Among many similar examples are TNF superfamily ligands on dendritic cell-derived exosome target TNF receptors of natural killer cells 69. The aggregation of human breast cancer cell (MDA-MB-231)-derived exosomes in the lung was due to the interaction between overexpressed exosomal integrin β4 and surfactant protein C of non-small cell lung cancer cells 159, 160.

**Figure 4:**
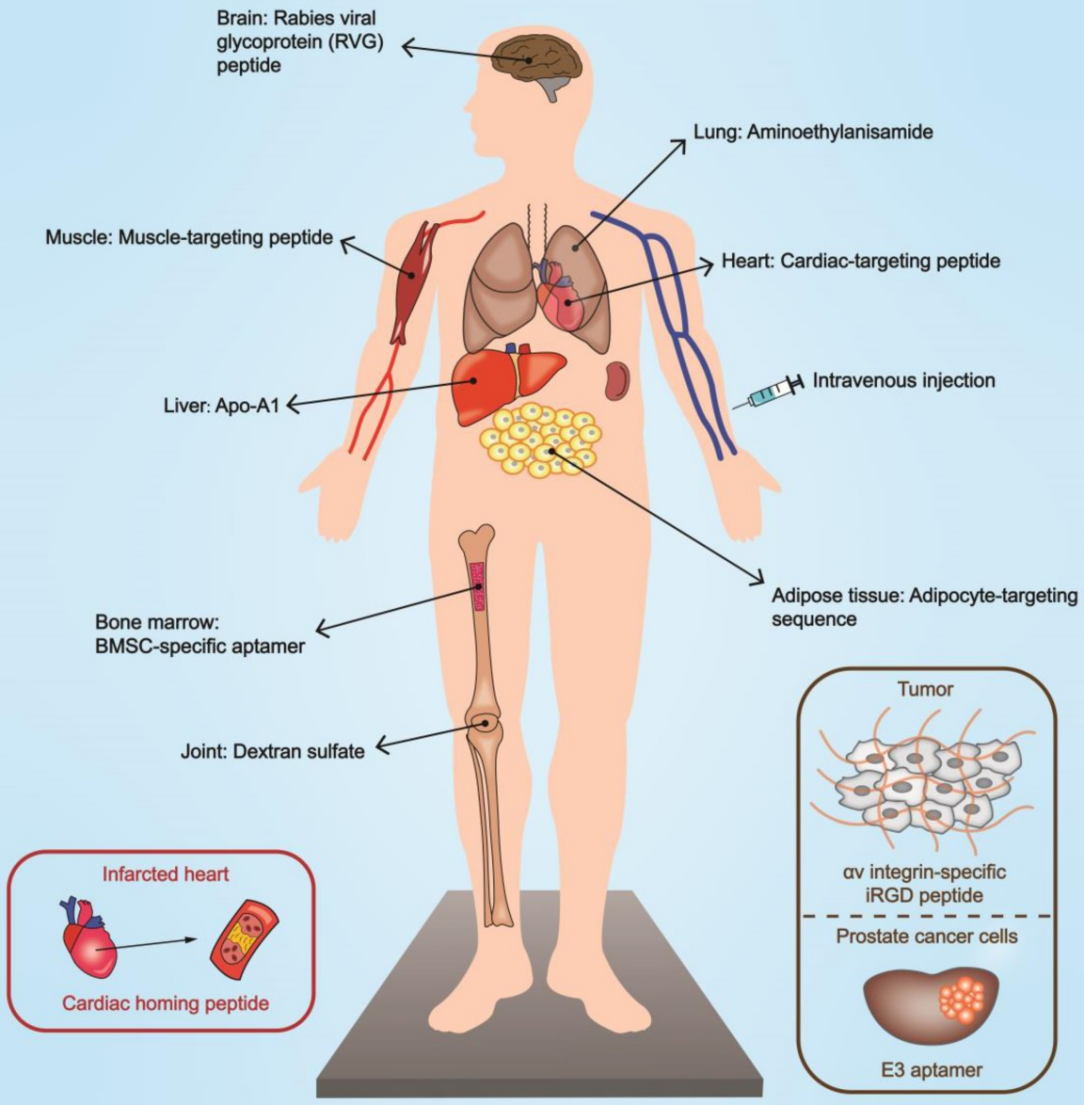
** Targeted delivery of EVs mediated by targeting moiety engineering.** Engineered EVs possess the ability to target specific organs and lesion sites, contributing to bioeffect optimization. The modes of functionalization include peptide, antibody, aptamer, and chemical substances. Specific modifications can selectively act on damaged or diseased tissues. BMSC, bone marrow mesenchymal stem cell.

One common trait in the examples mentioned above is that EVs are not modified. Although EVs have intrinsic targeting capacity based on natural recognition, they cannot accommodate incomputable disease patterns. Conferring an accurate orientation ability to EVs through appending suitable ligands represents an engineering modality that can endow EVs with desirable properties (Figure 4).

Genetic engineering is a critical approach to realizing the reprogramming of EVs 161. By constructing plasmids or lentiviral vectors encoding the targeting moiety to transfect/infect producer cells, it is possible to harvest functionalized EVs. Functional peptides with targeting ability can be fused to EV membrane proteins to mediate biological navigation. In this context, lamp2 (lysosomal-associated membrane protein 2), a well-characterized and abundantly expressed exosomal membrane protein, is a perfect site for modifications. RVG (rabies viral glycoprotein) peptides anchored onto lamp2b are capable of recognizing acetylcholine receptors on organs such as the brain 70 and kidneys 162. Other examples include αv integrin-specific iRGD peptide 12, cyclo(Arg-Gly-Asp-D-Tyr-Lys) peptide 163, and the muscle-specific peptide ligand 164 to hitch to tumor tissues, ischemic brain, and muscle respectively. The strategy of engineered glycosylation can contribute to maintaining the bioactivity of these well-designed peptides 165, and the phage display technology enables researchers to examine feasible peptides that can be attached to EVs 166.

Affibody is a scaffold protein consisting of 58 amino acids and is considered a targeting vector with excellent affinity 167. Liang et al. fused HER2-binding affibody to the extra-exosomal N terminus of human lamp2 to target colon cancer cells with high expression of HER2 67. Similarly, cytokines, such as interleukin 3 (IL-3), could be coated onto exosomes as a mediator to prime the IL3 receptor of chronic myelogenous leukemia blasts 168. As more potential loci are being elucidated, the binding sites are not restricted to lamp2b; CD63 (lamp3) 169 and platelet-derived growth factor receptor (PDGFR) 170 are also appropriate options.

The aptamer is a desirable ligand with a specific sequence that can decorate the surface of EVs and function in a dose-dependent manner within a specific range. It is a length of oligonucleotide sequence (DNA or RNA) in chemical nature, generated by multiple rounds of selection from a sequence library using the Cell-Internalization Systematic Evolution of Ligands by Exponential enrichment (SELEX) technique 171. The stabilized Schiff base formation is established by the reaction between aldehyde group modification on the 5'-end of the aptamer and the amino group on the membrane proteins of the exosome 172. For example, Sullenger and colleagues employed a designed aptamer (denoted as E3 aptamer) specific for prostate cancer cells 173. In another study, investigators employed solid-phase phosphoramidite chemistry to conjugate designed aptamers with diacyllipid tails, serving as a linker to facilitate exosome binding 174.

Engineered EVs are cast as the intermediaries to narrow the distance between two cell types, representing bidirectional targeting. Inspired by the chimeric antigen receptor T (CAR T) cell therapy concept, Li et al. adopted tumor antigen to stimulate dendritic cells, which prompted exosomes to present CD86 co-stimulating molecules and histocompatibility (MHC)-antigen complexes, serving as CAR. Besides, anti-CD3 was used to target CD3 on T cells and anti-epidermal growth factor receptor (EGFR) to target EGFR on cancer cells 68. This platform was denoted as synthetic multivalent antibodies retargeted exosomes (SMART-Exos) 170.

The interactions between chemical substances and receptors on specific membranes raise the possibility of targeted delivery of functionalized EVs by systemic administration. For example, linear 1,6-linked glucopyranoses with sulfate groups of dextran sulfate (DS) could be coupled with scavenger receptor class A (SR-A) of macrophages in treating rheumatoid arthritis (RA) 95. In addition, engineering modifications (e.g., cross-linking reaction) to specific lipids such as dioleoylphosphatidylethanolamine (DOPE) or distearoylphosphatidylethanolamine (DSPE) enable them to serve as a linker between the exosomal spheroid and targeting moiety. A DOPE-NHS (dioleoylphosphatidylethanolamine N-hydroxysuccinimide) linker was adopted in connecting cardiac homing peptide (CSTSMLKAC) and the exosomes to target infarcted heart specifically 175. Aminoethylanisamide (AA)-functionalized exosomes could be ligated to the sigma receptors on the lung cancer cells through the incorporation of DSPE-PEG-AA vector, as the terminal groups of PEGs are ideal sites for targeting moieties to link with 176.

Notably, the local delivery of EVs can increase the distribution in targeted organs with multiple auxiliary techniques. Ultrasound acts as a safety switch in controlling the activities of EVs. Microbubbles guided by ultrasound can interfere with the stability of the cell membrane and enhance penetrability of cavitation nuclei 180. We have previously elaborated the role of ultrasound-targeted microbubble destruction (UTMD) in the delivery of exosomes in refractory tissues 181. Sonication-sensitive materials can be adopted in the cascade reactions to realize local delivery. Sonosensitizer chlorin e6 (Ce6) can produce ROS under the effect of ultrasound irradiation, inducing the deshielding of PEG for further procedures 153. Physical connections have also found an increased application. Superparamagnetic iron oxide nanoparticles regulated by an external magnetic field adhered to exosomes through the Tf-TfR interaction with excellent targeting capability 71.

External carriers can also be adapted to facilitate the local delivery of EVs. Hydrogel, structured with a three-dimensional network, is an ideal candidate for EV-based delivery due to its excellent cellular biocompatibility, inherent antibacterial activity, and sustained release capability. The immobilization of exosomes can be achieved through the interaction between the accessional adhesive peptide PPFLMLLKGSTR of hydrogel and integrins on exosomal membranes 93. Bioactive scaffolds with high plasticity are desirable carriers as they display excellent competency 98.

### Promoting endocytosis and membrane fusion

Effective cellular uptake of EVs guarantees their biological functions. The primary form of endocytosis of EVs is pinocytosis, which can be subdivided into clathrin-dependent endocytosis (CDE), clathrin-independent endocytosis (CIE), and macropinocytosis (MP), among which CIE and MP are the commonest modalities 182-184. Similar conformation of lipids and shared molecular features increase the chances of membrane fusion. Engineered modifications to the membrane composition are also helpful in this context. In another example, the greatly enriched milk protein casein can self-assemble into micelles, and EVs mixed with casein showed enhanced cellular uptake 185. Similarly, Kang et al. incorporated amphiphilic phosphatidylcholine (PC) molecules into the phospholipid layer of reticulocyte-derived exosomes by incubation, vastly promoting their cellular internalization efficiency 186. Also, glycoengineering could selectively adjust the surface glycan constitution to alter the intensity of interactions between EVs and recipient cells 187. Noteworthy, we need to ensure that the newly introduced materials do not affect the original functions of EVs.

In the context of clinical translation, improved delivery minimizes the dosages of drugs and reduces side effects due to the toxicity to healthy cells. Vaccination is another important application of EVs, as unique nebulization treatments and inhalable modalities provide multi-protection compared with intramuscular injection 188. The immunogenicity and pathogenicity of EV vaccines should be precisely determined to achieve clinical standards. A multidimensional editing strategy without sacrificing bioactivity lays the foundation for improved delivery. EVs of autogenous origin tend to be the first choice with utmost adaptability and delivery efficiency for individual patients.

## Optimized manufacturing: formulation and characterization

**Figure 5:**
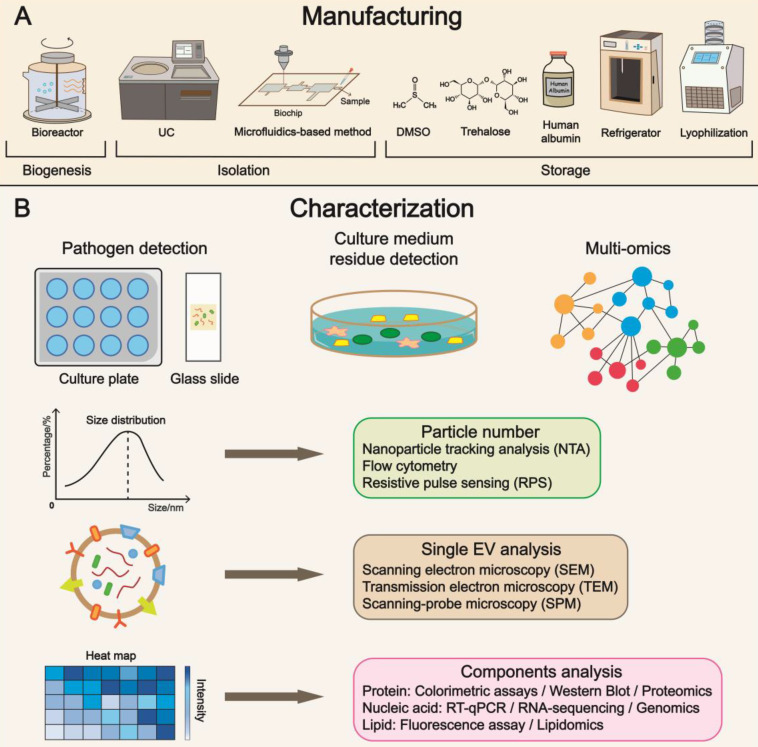
** Manufacturing, characterization, and subsequent clinical translation. (A)** Refinement in manufacturing is the focus of future research, covering a broad range of aspects from cell culture to storage. Multiple parameters such as temperature and additives have been identified to maintain the biological features of EVs. **(B)** Characterization enables precise evaluation of EVs and substantially expedites the progress of clinical translation. Pathogen and residue detections are approaches to examining purity and biosafety. The quality control of EV includes particle number, single EV, and components analysis using advanced techniques. UC, ultracentrifugation; DMSO, dimethyl sulfoxide.

With the recent progress in yield, isolation, cargo loading, and delivery, manufacturing issues have become increasingly important. In addition, quality control is a central issue that deserves further investigation (Figure 5) 189.

Bioreactor suspension cultures, in which the cells are dispersed, oscillated, and rotated in the medium, are widely applied in cell-based manufacturing. For the industrialization of EVs, the bioreactor-based culture system is preferred. Also, it is important to avoid using biological molecules from other species. For example, humanized materials, such as human growth factors and human serum albumin instead of bovine serum albumin, should be employed. More significantly, a system should be established to detect and prevent contamination during cultivation.

Suitable parameters for stable storage ensure the preservation of biosafety. Temperature is a determinant in EV storage. At the 4 °C-storage temperature, a reduction in the quantity and antibacterial capacity of EVs was observed within a short period, probably associated with aggregation, fusion, adsorption onto tube walls, and decomposition 190. Storage at -20 °C affects the EV size, whereas the total quantity is comparably stable 191. The temperature of -80 °C appears to be the most appropriate for long-term storage. Although multiple studies have reported controversial outcomes, for example, Brasier et al. denoted that -80 °C caused a 25% increase in diameter and an apparent decline in the zeta potential of EVs 192, the cold chain logistics industry brings new opportunities to -80 °C setting. The development of temperature control relies on the mechanical engineering industry with collective efforts from multiple fields.

Phosphate-buffered saline (PBS) is used for stabilizing EVs, protecting them from denaturation or degradation, and maintaining pH and electrolytes at normal levels. Trehalose has been broadly utilized as a protein stabilizer and cryoprotectant, and its nontoxicity and low moisture absorption are suitable for EV preservation. Bosch et al. revealed that particle size distribution narrowed and increased the concentration of EVs per milligram of protein after trehalose treatment 193. The recovery rate of EVs could be improved in PBS supplemented with human albumin and trehalose at -80 °C 194. Another practical method includes dimethyl sulfoxide (DMSO) cryopreservation 195. To sum up, flexible adjustments of additives may generate surprisingly positive outcomes. Lyophilization is an epoch-making technology in the pharmaceutical industry that greatly benefits transportation. The stability of colloidal EVs (lyophilized EV formulations) is significantly enhanced within sucrose or potassium phosphate buffer, while the import of traces of poloxamer 188 would augment this effect 196. Chemical substances with amplification effect in storage capacity need to be discovered, ranging from native to synthesized compounds.

Multidimensional characterization is the major form of quality control with precision through cross-validation. The entities include producer cells (e.g., phenotypes, vesiculation capacity), isolated EVs (e.g., single-EV analysis, batch heterogeneity), and potential contaminations (e.g., bacteria, viruses, residua of the mediums, reagents) 189. Testing tools, such as versatile microfluidic chips and advanced nano-flow cytometry for single-EV analysis, are being constantly developed 108, 197. Lu et al. reported a microarray platform constituted by a series of membrane protein-specific antibodies and microscopic hyperspectral imaging to distinguish EVs. Moreover, by constructing molecular fingerprints, the range of characterization is not limited to EVs, which also incorporates the donor cells 198. The aspects of quality control include bioactivity, physiological status, purity, and homogeneity. Herein, detection approaches are summarized based on the published literature. A systematic and standardized evaluation system remains to be established, forming consensus in the EV field.

## Application of the EV manipulating strategies in clinical trials

Currently, a multitude of clinical trials have been designed and conducted. Importantly, native EVs (without extra modifications) of diverse origins have found utility in various diseases. To overcome the limitation of the low yield of EVs from autologous or allogenic cells, EVs from HEK293 cells are explored in clinical trial settings (NCT05375604 and NCT04592484). EVs from plant have been also tested in cancer therapy (NCT01294072). Beyond the donor cell source optimization, the strategies of loading have also been successfully adopted in EV functionalization. A sequence of trials sponsored by Codiak BioSciences Inc. have been attempting to confirm that cargoes including STimulator of InterferoN Genes (STING) agonists (NCT04592484) and STAT6-targeting antisense oligonucleotide (ASOs) (NCT05375604) could be loaded into EVs to exert antitumor effects. Our group has designed the first-in-human study (NCT05043181), in which bone marrow-derived MSCs with LDL receptor (LDLR) overexpressed served as parental cells to ensure efficient mRNA loading. Further, with the goal of bolstering delivery efficacy, in an open-label study, researchers would utilize focused ultrasound to facilitate the delivery of EVs derived from amniotic fluid to the subgenual target for patients with refractory depression (NCT04202770). In summary, the strategies toward high yield, facile isolation, improved delivery and efficient loading are just being recognized in the clinical trials (Table 4).

As our knowledge of EVs deepens, we are faithful that a rational design of clinical trials and a throughout analysis of the achieved results will collectively contribute to the clinical translation of EVs. Going forward, we suggest the researchers to give more consideration to the strategy of improved delivery (e.g., decoration of ligands for targeting) when designing a clinical trial.

## Conclusion and prospective

EV-based therapy has a bright prospect attributed to the unique structure and composition of EVs. However, the insufficient yield of EVs has restricted their clinical translation. Researchers have made enormous progress with significant advances in mass production, purification, cargo loading, and EV delivery (Figure 1). We believe a rational combination of these strategies would promote the clinical translation for specific diseases. A systematic comparison of the parallel methods for a specific purpose is a prerequisite to choosing the best one. Then, as the strategies might conflict, a disease/patient-specific combination strategy should be explored by small-scale experiments before manufacturing. Finally, the most optimized strategy should be chosen for manufacturing.

Standardization of protocols is crucial for manufacturing 199. Furthermore, attention must be focused on quality control and the long-term storage of EVs. With technical support from electrical automation and machine learning, an artificially intellectualized workstation is expected, holding tremendous potential to be mined.

Strategies in EV manufacturing and quality control aspects should be performed in a coordinated manner, contributing to the ultimate goal of EV-based therapy. A sequential pattern of “Secretion-Isolation-Purification-Encapsulation/Modulation-Characterization/Quality control-Delivery-Evaluation” could be a conceptual roadmap for clinical translation of EV-based therapy. Manufacturing dominates the steps before “Delivery” while the rest of the flow is assigned to medical workers. Future direction lies in appending branches to this patient-centered route based on feedback response, turning it into a multi-threaded network. Delightedly, a vast number of scientists are already engaged in.

For commercialization and clinical translation of EVs, advanced animal experiments and more clinical trials must be conducted to detect probable adverse effects. Scientists should also execute a comprehensive preclinical examination of pharmacokinetics for EV-based products. On the strength of these strategies, we firmly believe that the pace of clinical translation for EVs can be accelerated markedly.

## Figures and Tables

**Table 1:** Derivations of EVs and their applications

Derivation	Merits	Drawbacks	Application Scenarios	Ref.
Differentiated cells from induced pluripotent stem cells	-Artificial induction with selectivity -Stable sources of EVs (differentiate into various cell types) -Consecutive production of autologous EVs -Bypass ethical issues	-Underlying genetic defects -Tumorigenicity -Heterogeneity due to inconsistency of differentiation -Incomplete mechanisms	-Treatment of heart failure and myocardial infarction -Regenerative medicine -Drug tests and screening -Disease modeling -Applicable to precision and personalized medicine	63, 64
Cancer Cells	-Characteristic biomarkers and bioactive molecules -Separation from bio-fluids -Benefit from the development of detection technology	-Limited protein profiling for derived EVs -Incomplete understanding of the mechanisms -Safety concerns about inducing immunologic tolerance and negative immune regulation	-Biomarkers for diagnosis -Engagement in cancer development -Therapeutic function	65, 66
**Established cell line**				
HEK293T/AML12/RAW264.7	-Guaranteed source -mass production capacity for industrialization -Standardized protocols (applicability and universality)	-May contain carcinogenic ingredients	-Therapeutic effect -Anti-tumor features	67
**Immune cell**				
Dendritic cell	-Alter the properties under feasible stimulus (antigens/cytokines), -Expression of multiple immune-associated molecules -Activator of immune responses	-Heterogeneity in different stages (deficiency of MHC-II and CD86 in immature stage)	-Chimeric antigen receptor T cell therapy -Induce the apoptosis of cancer cells -Activate natural killer cells -Treatment of Alzheimer's disease	68-70
Neutrophil	-Tumor-targeting ability -Intrinsic inflammation-tropism -Outstanding blood-brain barrier crossing capability	-Latent pathogenicity	-Suppress tumor growth -Therapy for glioma -Induce chronic obstructive pulmonary disease and bronchopulmonary dysplasia	71-73
Macrophages	-Intrinsic inflammatory chemotaxis -Expression of specific receptors -Involve multiple cytokines	-Heterogeneity in functions and phenotypes of different sources	-Alleviate rheumatoid arthritis -Management of atherosclerosis- -Remodel the tumor microenvironment -Enhance antitumor effect	17, 74-76
**Blood**				
Plasma	-Accessibility -High bio-safety -Wide distribution *in vivo* -Maneuverability	-Difficulty in precise separation	-Alleviate spontaneous hypertension -Biomarker in the diagnosis of multiple diseases	77-79
Red blood cells	-Biosafety (devoid of nuclear and mitochondrial DNA) -Abundant resources in the blood bank -Accessibility -Brain targeting ability	-Underlying side effect -Various factors affect half-life and shelf life	-Therapy for Parkinson's disease -Drug delivery	56, 80, 81
Platelet	-Tumor-homing property -Apheresis facilitates the collection procedure -Enrichment of various bioactive molecules -Nuclear-free (minimized teratogenicity)	-Heterogeneity among donors -Probable pathogen contamination	-Chemo-dynamic/Photothermal therapy for cancer -Regenerative medicine -Drug delivery	82, 83
**Neural cells**				
Microglia	-Rich in trophic factors	-Difficulty in cell acquisition	-Prevent ischemia-reperfusion injury -Enhance neurogenesis and neuroprotective effect -Cure traumatic brain injury	84, 85
Astrocytes	-Most abundant neuroglial cells	As above	-Mitigate neurotoxicity in Alzheimer's disease	35
**Adipose tissue**				
Brown adipose tissue	-Promote energy metabolism -Rich in mitochondria	-Limited supplement (primarily in infants or neonatal mammals)	-Anti-obesity -Alleviate metabolic syndrome	86
White (visceral) adipose tissue	-Ability to cross the placenta -Effective uptake by vascular endothelial cells	-Relatively finite sources	-Related to fetal cardiac dysfunction -Management of colitis	87, 88
**Mesenchymal stem cell**				
Classification as below	-Self-renewal capacity -Multiple differentiation potential -Strong reproductive activity *in vitro* -Immunoregulation ability	-Safety concerns between donor and recipient -Require expansion	Varying from cell types as below	89, 90
Bone marrow mesenchymal stem cells	-Various bio-signal molecules	-Discrepancy in clinical trials -Limited knowledge of working principles	-Tissue engineering -Treatment of osteoarthritis -Neuro-degenerative diseases	15, 91, 92
Human umbilical cord mesenchymal stem cells	-Phenotypes change under stimuli -Primitiveness -lower reject reaction	-Relatively limited source -Ethical issue	-Treatment of spinal cord injury -Accelerate chronic diabetic wound healing	93, 94
Adipose-derived stem cells	-Contain Various cytokines -Broad distribution in the human body	-Heterogeneity in methods of administration -Biosafety *in vivo* under evaluation	-Remodel inflammatory microenvironment -Tissue repair and reconstruction -Treatment of rheumatoid arthritis/intervertebral disc degeneration/tendon lacerations	95-99

**Table 2:** Summary of the isolation methods for EVs

Isolation methods	Operating principles	Pros	Cons
Differential centrifugation	Adjust centrifugal force via altering RPM; suspension or sediment in line with density	Widely applied and relatively standardized protocols; volume production; low cost of extraction in the condition of existing instruments	High expense of stationary instruments; cost of time and manpower; protein aggregation
Gradient ultracentrifugation	Form density gradient through medium; constituents with various densities lie in different positions	Highly purified; handle easily with protocols	High cost of stationary equipment; time-consuming; low productivity; latent changes under mechanical force
Size-exclusion chromatography	Sieve pores through crosslinking of polymers; separation on the basis of sizes	High purity; maintenance of original structure; applicable to various derivations	Relatively high expense of devices; requirement of extra procedures
Ultrafiltration	Filter through membranes of different apertures; isolation in dependence on the sizes	Great portability without costly equipment; fast operation; capable of mass production	Moderate purity; membrane obstructions and trapping; potential destructive effects of shear stress
Extrusion	Utilize extruder and designed membranes; sized-based technique	Fast preparation; high yield	Additional operations of ultracentrifugation; output as exosome-mimetics
Precipitation	Harness water-excluding polymers to remodel the solubility	Scalability; simple and practicable	Coprecipitation with proteins and polymers harming purity; requisite procedures of clean-up; contaminants may interfere downstream analysis
Immunoaffinity	High affinity between immobilized antibodies (ligands) and exosomal receptors	Highly specificity; high purity; lessened contaminants	High cost of antibodies; narrow scope of application (cell-free samples with distinct markers); elution step may impair the original states
Microfluidics-based method	Construct microfluidic channel systems to filter EVs; established on different principles including size, density and immunoaffinity	Focus of research with tremendous room for upgradation; high recovery rate; achievable automatic operating system; portability; development of biochips boosts this technique	Low sample capacity; require standardization of operational procedures
Commercial kits	Multiple product types based on the above-mentioned principles	Portability without stationary apparatus; apt to commercialization; cost-effective in the absence of stationary instruments	Low purity; different efficiencies of disparate products; relatively expensive in specific circumstances

**Table 3:** Mechanisms of targeting in EVs

Ligand	Mechanisms	Derivation of EVs	Receptor	Recipient cell/Organ	Ref.
**Peptide**					
Muscle-targeting peptide	Conjugated with CD63 through CP05	Murine myotubes	NA	Muscle	166
Neuron-specific rabies viral glycoprotein peptide (YTIWMPENPRPGTPCDIFTNSRGKRASNG)	Fused to Lamp2b	Dendritic cells	Acetylcholine receptor	Brain	70
αv integrin-specific iRGD peptide (CRGDKGPDC)	Fused to Lamp2b	Immature dendritic cells	αv integrin	Tumor tissues (MDA-MB-231 tumor)	12
Ischemic myocardium-targeting peptide (CSTSMLKAC)	Fused to Lamp2b	Mesenchymal stem cell	NA	Ischemic myocardium	177
Cardiac-targeting peptide (APWHLSSQYSRT)	Fused to Lamp2b	HEK 293 cells	NA	Heart	149
Cardiac homing peptide (CSTSMLKAC)	Conjugated with exosomes through a DOPE-NHS linker	Cardiosphere-derived stem cells	NA	Infarcted heart	175
Cyclo(Arg-Gly-Asp-D-Tyr-Lys) peptide	Click chemistry	Bone marrow-derived mesenchymal stromal cell	Integrin αvβ3	Reactive cerebral vascular endothelial cells of ischemic brain	163
Adipocyte-targeting sequence (CKGGRAKDC)	Fused to Lamp2b	HEK293T	Prohibitin	Adipocyte	121
**Antibody**					
A33 antibody	Antigen-antibody reaction	LIM1215 cells	A33 antigen	Colon cancer cells	178
Anti-CD3	Connected with exosome through DSPE-PEG-NHS linker	Tumor antigen-stimulated dendritic cells	CD3	T cells	68
Anti-EGFR	As above	As above	EGFR	B16-OVA cancer cells	As above
**Antibody**					
Her2 binding affibody (VDNKFNKEMRNAYWEIALLPNLNNQQKRAFIRSLYDDPSQSANLLAEAKKLNDAQAPK)	Fused to the extra-exosomal N terminus of human Lamp2	HEK293T cells	Her2	5-FU-resistant HCT-116 colon cancer cells	67
**Aptamer**					
Bone marrow mesenchymal stem cell (BMSC)-specific aptamer (5′-ACGACGGTGATATGTCAAGGTCGTATGCACGAGTCAGAGG-3′)	Schiff base reaction	Bone marrow stromal cell	NA	BMSC	172
E3 aptamer	Thiol-maleimide cross-linking reaction	HEK293T cells	NA	Prostate cancer cells	179
**Cytokines**					
IL-3	Fused to Lamb2b	HEK293T cells	IL3 receptor	Chronic myelogenous leukemia cells	168
**Chemokine receptors**					
CD44	Intrinsic inflammation-tropism capability	M2 macrophage	E-selectin	Inflammatory endothelial cells	158
VLA4	As above	As above	VCAM1	As above	As above
LFA1	As above	As above	ICAM1	As above	As above
**Others**					
Aminoethylanisamide	Incorporation of AA-PEG vector moiety	Primary bone-marrow derived macrophages	Sigma receptor	Lung cancer cells	176
Apo-A1	Fused to CD63	HEK293T cells	SR-B1	HepG2 cells	169
Dextran sulfate	Click chemistry	Adipose-derived stem cells	SR-A	Activated macrophages in inflamed joints	95
Integrin β4	Overexpression of integrin β4	Breast cancer cells (MDA-MB-231)	Surfactant protein C	Non-small cell lung cancer cells	160
Folic acid	Attached folic acid covalently	Colostrum powder	Folate receptor α	Lung cancer cell line A549	52

CD, cluster of differentiation; Lamp2b, Lysosomal-associated membrane protein 2b; DOPE-NHS, dioleoylphosphatidylethanolamine N-hydroxysuccinimide; DSPE-PEG-NHS, phospholipid-polyethylene glycol-succinimide; EGFR, epidermal growth factor receptor; Her2, human epidermal growth factor receptor 2; BMSC, bone marrow mesenchymal stem cell; IL-3, interleukin 3; VLA4, very late antigen 4; VCAM1, vascular cell adhesion molecule 1; LFA1, lymphocyte function-associated antigen 1; ICAM1, endothelial intercellular adhesion molecule 1; AA-PEG, aminoethylanisamide-polyethylene glycol; SR-B1, scavenger receptor class B type 1; SR-A, scavenger receptor class A; NA, not available.

**Table 4:** Representative clinical trials of EVs

Trial identifier	Source (product name)	Modifications	Study aim	Recruitment status
NCT04652531	Autologous Serum	-Unmodified	Treatment for venous trophic lesions	Recruiting
NCT05499156	Human placenta MSC	-Unmodified	Safety evaluation of EVs for treating perianal fistula in patients with Crohn's disease	Active, not recruiting
NCT04276987	Allogenic adipose MSC	-Unmodified	Treatment for severe novel coronavirus pneumonia	Completed
NCT04202770	Amniotic fluid	-Delivery aided by focused ultrasound	Therapy of refractory depression, anxiety, and neurodegenerative dementias	Suspended
NCT05043181	Bone marrow MSC	-Loaded with LDL receptor mRNA	Treatment of homozygous familial hypercholesterolemia	Not yet recruiting
NCT03608631	Mesenchymal stromal cells	-Loaded with small interference RNA against KrasG12D	Treating metastatic pancreas cancer with KrasG12D mutation	Recruiting
NCT01294072	Plant	-Loaded with curcumin	Delivering curcumin to normal and colon cancer tissue	Recruiting
NCT05375604	HEK 293 cells (exoASO-STAT6)	-Loaded with STAT6-targeting ASOs	Verifying the antitumor effect of exoASO-STAT6	Recruiting
NCT04592484	HEK 293 cells (ExoSTING)	-Loaded with STING agonist	Treatment for patients with certain solid tumors	Active, not recruiting
NCT01159288	Dendritic cell	-Loaded with tumor antigen	Vaccination for unresectable NSCLC patients	Completed

MSC, mesenchymal stem cells; LDL, low-density lipoprotein; STAT6, signal transducer and activator of transcription 6; ASO, antisense oligonucleotide; STING, STimulator of InterferoN Genes; NSCLC, non-small-cell lung cancer.
